# Supplementary material for: Anthropogenic Influence on Moth Populations: A Comparative Study in Southern Sweden
Source: Insects. 2023 Aug 11;14(8):702. doi: 10.3390/insects14080702 (PMC10455763; doi:10.3390/insects14080702)
Supplement: Supplementary file 1 [file insects-14-00702-s001.zip › insects-2537489-supplementary.pdf]

Species name	Taxonomic family	Red list category	Year new to Småland	Year new to Västergötland	Individuals in Borås trap	Individuals in Kalmar trap	Color pattern variation	Length of flight period	Habitat preference	Host plant specificity	Overwintering stage
Abraxas grossulariata	Geometridae		1900	1900			2	6	Generalist	2	Larva
Abrostola tripartita	Noctuidae		1900	1900	6	1	0	7	Generalist	2	Pupa
Abrostola triplasia	Noctuidae		1900	1900	10	1	0	10	Generalist	2	Pupa
Acronicta aceris	Noctuidae		1900	1900		1	0	8	Forest	3	Pupa
Acronicta auricoma	Noctuidae		1900	1900	15	1	1	4	Generalist	3	Pupa
Acronicta cinerea	Noctuidae		1900	1900			1	9	Open	3	Pupa
Acronicta cuspis	Noctuidae		1900	1900	2		0	8	Forest	2	Pupa
Acronicta leporina	Noctuidae		1900	1900			1	9	Forest	3	Pupa
Acronicta megacephala	Noctuidae		1900	1900	8		0	6	Generalist	3	Pupa
Acronicta menyanthidis	Noctuidae		1900	1900			0	8	Generalist	3	Pupa
Acronicta psi	Noctuidae		1900	1900	39	2	0	9	Generalist	3	Pupa
Acronicta rumicis	Noctuidae		1900	1900	41	7	1	12	Generalist	3	Pupa
Acronicta tridens	Noctuidae		1900	1900			0	7	Generalist	3	Pupa
Actinotia polyodon	Noctuidae		1900	1900		1	0	8	Open	2	Pupa
Agriopis marginaria	Geometridae		1900	1900		3	1	3	Generalist	3	Pupa
Agrochola circellaris	Noctuidae		1900	1900	5	7	0	6	Generalist	3	Egg
Agrochola helvola	Noctuidae		1900	1900	11		0	6	Forest	3	Egg
Agrochola litura	Noctuidae		1900	1900			0	4	Forest	3	Egg
Agrochola lota	Noctuidae		1900	1900			0	6	Forest	3	Egg
Agrotis clavis	Noctuidae		1900	1900	21	33	1	5	Generalist	3	Larva
Agrotis exclamationis	Noctuidae		1900	1900	76	40	1	16	Generalist	3	Larva
Agrotis ipsilon	Noctuidae		1900	1900	2		1	16	Generalist	3	Larva
Agrotis segetum	Noctuidae		1900	1900		5	2	17	Generalist	3	Larva
Agrotis vestigialis	Noctuidae		1900	1900			1	6	Open	3	Larva
Alcis jubata	Geometridae		1900	1900			2	7	Generalist	3	Larva
Alcis repandata	Geometridae		1900	1900	84		2	7	Generalist	3	Larva
Allophyes oxyacanthae	Noctuidae		1900	1900	55	23	0	6	Generalist	2	Egg
Alsophila aescularia	Geometridae		1900	1900		5	0	3	Forest	3	Pupa
Ammoconia caecimacul	Noctuidae		1900	1900	1		0	7	Generalist	3	Egg
Amphipoea oculea	Noctuidae		1900	1900	17	10	1	7	Generalist	3	Egg
Amphipyra pyramidea A	Noctuidae		1900	1900	6	47	0	6	Generalist	3	Egg
Anaplectoides prasina	Noctuidae		1900	1900	21		0	7	Forest	3	Larva
Anarta myrtilli	Noctuidae		1900	1900			0	11	Open	2	Pupa
Anarta trifolii	Noctuidae		1900	1900			1	13	Generalist	3	Pupa
Angerona prunaria	Geometridae		1900	1900	8		2	4	Generalist	3	Larva
Antitype chi	Noctuidae		1900	1900			0	5	Open	3	Egg
Apamea crenata	Noctuidae		1900	1900	1		2	7	Generalist	3	Larva
Apamea furva	Noctuidae		1900	1900		2	0	6	Generalist	3	Larva
Apamea lateritia	Noctuidae		1900	1900	4		0	6	Open	3	Larva
Apamea monoglypha	Noctuidae		1900	1900	19	16	1	10	Generalist	3	Larva
Apamea oblonga	Noctuidae		1900	1900			1	5	Generalist	3	Larva
Apamea sordens	Noctuidae		1900	1900	1		0	6	Generalist	3	Larva
Aplocera plagiata	Geometridae		1900	1900		14	0	13	Open	2	Larva
Apoda limacodes	Limacodidae		1900	1900	1	4	0	8	Forest	2	Pupa
Archiearis parthenias	Geometridae		1900	1900			1	3	Forest	3	Pupa
Arctia aulica	Erebidae		1900	1900			0	2	Open	3	Larva
Arctia caja	Erebidae		1900	1900	19	1	2	9	Generalist	3	Larva
Arctia plantaginis	Erebidae		1900	1900			1	6	Open	3	Larva
Arichanna melanaria	Geometridae		1900	1900	10		1	7	Generalist	2	Larva
Atolmis rubricollis	Erebidae		1900	1900	18		0	4	Open	3	Pupa
Autographa gamma	Noctuidae		1900	1900	23	15	0	13	Generalist	3	Larva
Autographa jota Autogr	Noctuidae		1900	1900			0	5	Generalist	3	Larva
Autographa pulchrina	Noctuidae		1900	1900	5		0	6	Generalist	3	Larva
Axylia putris	Noctuidae		1900	1900	2		0	14	Generalist	3	Pupa
Biston betularia	Geometridae		1900	1900	42		2	10	Generalist	3	Pupa
Brachylomia viminalis	Noctuidae		1900	1900	12		0	8	Forest	3	Egg
Bupalus piniaria	Geometridae		1900	1900	7		2	10	Forest	2	Pupa
Cabera exanthemata	Geometridae		1900	1900	42	2	0	10	Generalist	3	Pupa
Cabera pusaria	Geometridae		1900	1900	123	9	0	10	Generalist	3	Pupa
Calliteara abietis	Erebidae		1900	1900	1		1	5	Forest	2	Larva
Campaea margaritaria	Geometridae		1900	1900	70	1	0	4	Generalist	3	Larva
Camptogramma bilinea	Geometridae		1900	1900	13	17	0	8	Open	2	Larva
Caradrina clavipalpis	Noctuidae		1900	1900		6	1	18	Generalist	3	Pupa
Caradrina morpheus	Noctuidae		1900	1900	126	69	1	9	Generalist	3	Larva
Carsia sororiata	Geometridae		1900	1900			2	7	Generalist	2	Egg
Catarhoe cuculata	Geometridae		1900	1900		2	0	4	Open	2	Pupa
Catocala fraxini	Erebidae		1900	1900	6		1	4	Generalist	3	Egg
Catocala nupta	Erebidae		1900	1900			1	6	Forest	2	Egg
Ceramica pisi	Noctuidae		1900	1900	2		1	7	Generalist	3	Pupa
Cerapteryx graminis	Noctuidae		1900	1900	36	26	0	9	Generalist	3	Egg
Cerastis rubricosa	Noctuidae		1900	1900	22	1	0	6	Forest	3	Pupa
Cerura vinula	Notodontidae		1900	1900			1	9	Generalist	3	Pupa
Charanyca trigrammica	Noctuidae		1900	1900		5	0	6	Open	2	Larva
Charissa obscurata	Geometridae		1900	1900			0	6	Generalist	3	Larva
Chiasmia clathrata	Geometridae		1900	1900	8	14	2	11	Open	3	Pupa
Chlorissa viridata	Geometridae		1900	1900	1		0	7	Open	2	Pupa
Chloroclysta miata	Geometridae		1900	1900	4		0	15	Generalist	3	Larva
Chloroclysta siterata	Geometridae		1900	1900	16	1	1	13	Generalist	3	Larva
Cidaria fulvata	Geometridae		1900	1900		19	0	4	Open	2	Egg
Cirrhia icteritia	Noctuidae		1900	1900	18	15	1	6	Forest	3	Egg
Cleora cinctaria	Geometridae		1900	1900		1	1	4	Generalist	3	Pupa
Cleorodes lichenaria	Geometridae		1900	1900	2	1	1	6	Generalist	3	Larva
Clostera curtula	Notodontidae		1900	1900	1		0	9	Forest	3	Pupa
Clostera pigra	Notodontidae		1900	1900	1	1	0	10	Forest	3	Pupa
Colocasia coryli	Noctuidae		1900	1900	4		1	4	Forest	3	Pupa
Colostygia pectinataria	Geometridae		1900	1900	7	1	0	7	Generalist	2	Larva
Colotois pennaria	Geometridae		1900	1900	84	4	1	5	Forest	3	Egg
Conistra rubiginea	Noctuidae		1900	1900	3	1	0	10	Generalist	3	Imago
Conistra vaccinii	Noctuidae		1900	1900	136	31	2	10	Generalist	3	Imago
Coscinia cribraria	Erebidae		1900	1900			1	4	Forest	3	Larva
Cosmia trapezina	Noctuidae		1900	1900	44	43	2	8	Generalist	3	Egg
Cosmorhoe ocellata	Geometridae		1900	1900	64	10	0	11	Generalist	2	Larva
Cossus cossus	Cossidae		1900	1900			0	5	Generalist	3	Larva
Crocallis elinguaria	Geometridae		1900	1900	9	11	1	5	Generalist	3	Larva
Cybosia mesomella	Erebidae		1900	1900	90		1	6	Open	3	Larva
Cyclophora albipunctata	Geometridae		1900	1900	7		0	8	Generalist	2	Pupa
Cyclophora pendularia	Geometridae		1900	1900		1	0	6	Forest	2	Pupa
Cyclophora punctaria	Geometridae		1900	1900	2	2	1	10	Forest	3	Pupa
Dasypolia templi	Noctuidae		1900	1900			0	7	Generalist	3	Imago
Deilephila elpenor	Sphingidae		1900	1900	4		0	10	Generalist	3	Pupa
Deilephila porcellus	Sphingidae		1900	1900	2	1	0	11	Open	2	Pupa
Deltote uncula	Noctuidae		1900	1900	4		0	6	Generalist	2	Pupa
Dendrolimus pini	Lasiocampidae		1900	1900	3		1	5	Forest	2	Larva
Diachrysia chrysitis Diac	Noctuidae		1900	1900	8	1	0	10	Generalist	3	Larva
Diacrisia sannio	Erebidae		1900	1900	14		0	7	Open	3	Larva
Diaphora mendica	Erebidae		1900	1900	2	10	0	3	Open	3	Pupa
Diarsia brunnea	Noctuidae		1900	1900	266	1	0	7	Generalist	3	Larva
Diarsia mendica	Noctuidae		1900	1900	11		2	4	Generalist	3	Larva
Diarsia rubi Diarsia flori	Noctuidae		1900	1900	3	1	0	7	Generalist	3	Pupa
Dicycla oo	Noctuidae	NT	1900	1900		2	1	6	Forest	2	Larva
Diloba caeruleocephala	Noctuidae		1900	1900		5	0	4	Forest	3	Egg
Drepana falcataria	Drepanidae		1900	1900	44		1	9	Forest	3	Pupa
Drymonia ruficornis	Notodontidae		1900	1900		4	0	6	Forest	2	Pupa
Dryobotodes eremita	Noctuidae		1900	1900		3	1	4	Forest	2	Egg
Dypterygia scabriuscula	Noctuidae		1900	1900		3	0	6	Forest	2	Pupa
Dysstroma citrata	Geometridae		1900	1900	92		2	8	Generalist	3	Egg
Dysstroma truncata	Geometridae		1900	1900	44		2	14	Generalist	3	Larva
Earias clorana	Nolidae		1900	1900			0	9	Generalist	2	Pupa
Ecliptopera silaceata	Geometridae		1900	1900	39		0	11	Generalist	2	Pupa
Ectropis crepuscularia	Geometridae		1900	1900	7		2	12	Generalist	3	Pupa
Eilema lurideola	Erebidae		1900	1900	1	42	0	6	Generalist	3	Larva
Eilema lutarella	Erebidae		1900	1900	25	8	0	3	Open	3	Larva
Electrophaes corylata	Geometridae		1900	1900	1		1	7	Forest	3	Pupa
Ematurga atomaria	Geometridae		1900	1900			2	12	Generalist	3	Pupa
Enargia paleacea	Noctuidae		1900	1900	21	1	0	7	Generalist	3	Egg

Endromis versicolora	Endromidae		1900	1900	3		0	6	Forest	3	Pupa
Ennomos erosaria	Geometridae		1900	1900	34	29	0	11	Forest	2	Egg
Entephria caesiata	Geometridae		1900	1900			1	6	Forest	2	Larva
Epione repandaria	Geometridae		1900	1900	4		0	8	Forest	2	Egg
Epione vespertaria	Geometridae		1900	1900	11		0	6	Forest	3	Egg
Epirranthis diversata	Geometridae		1900	1900			1	9	Forest	3	Pupa
Epirrhoe alternata	Geometridae		1900	1900	111	161	1	8	Generalist	2	Pupa
Epirrhoe galiata	Geometridae		1900	1900			0	7	Generalist	2	Pupa
Epirrhoe tristata	Geometridae		1900	1900	16		1	8	Open	2	Pupa
Eriogaster lanestris	Lasiocampidae		1900	1900			0	3	Generalist	3	Pupa
Euchoeca nebulata	Geometridae		1900	1900			0	6	Forest	2	Pupa
Euclidia glyphica	Erebidae		1900	1900			0	7	Open	2	Pupa
Euclidia mi	Erebidae		1900	1900			0	5	Open	3	Pupa
Eulithis populata	Geometridae		1900	1900	156		2	10	Generalist	3	Egg
Eulithis prunata	Geometridae		1900	1900	7	2	1	8	Generalist	2	Egg
Eulithis testata	Geometridae		1900	1900	29	1	1	7	Generalist	3	Egg
Eupithecia abietaria	Geometridae		1900	1900			1	6	Forest	2	Pupa
Eupithecia absinthiata	Geometridae		1900	1900			1	7	Generalist	3	Pupa
Eupithecia centaureata	Geometridae		1900	1900		11	0	13	Open	2	Pupa
Eupithecia icterata	Geometridae		1900	1900	11		2	8	Open	3	Pupa
Eupithecia intricata	Geometridae		1900	1900			1	4	Generalist	2	Pupa
Eupithecia lanceata	Geometridae		1900	1900			0	5	Forest	2	Pupa
Eupithecia pusillata	Geometridae		1900	1900	11		1	8	Generalist	2	Egg
Eupithecia satyrata	Geometridae		1900	1900			1	7	Generalist	3	Pupa
Eupithecia simpliciata	Geometridae		1900	1900			1	7	Generalist	2	Pupa
Eupithecia subfuscata	Geometridae		1900	1900			1	7	Generalist	3	Pupa
Eupithecia succenturiata	Geometridae		1900	1900			1	12	Open	3	Pupa
Eupithecia vulgata	Geometridae		1900	1900			1	8	Generalist	3	Pupa
Euplexia lucipara	Noctuidae		1900	1900	7		0	9	Forest	3	Pupa
Eupsilia transversa	Noctuidae		1900	1900	6	22	1	10	Generalist	3	Imago
Eurois occulta	Noctuidae		1900	1900	9	1	1	10	Forest	3	Larva
Euxoa nigricans	Noctuidae		1900	1900	1	4	0	7	Generalist	3	Larva
Euxoa obelisca	Noctuidae		1900	1900		5	0	7	Open	3	Larva
Euxoa tritici	Noctuidae		1900	1900	1	8	2	8	Generalist	3	Larva
Falcaria lacertinaria	Drepanidae		1900	1900	31		1	9	Forest	3	Pupa
Furcula furcula	Notodontidae		1900	1900	4		0	8	Generalist	3	Pupa
Gandaritis pyraliata	Geometridae		1900	1900	6	2	1	7	Forest	2	Egg
Geometra papilionaria	Geometridae		1900	1900	6		0	7	Generalist	3	Larva
Gnophos obfuscata	Geometridae		1900	1900			0	7	Generalist	3	Larva
Graphiphora augur	Noctuidae		1900	1900			0	7	Generalist	3	Larva
Griposia aprilina	Noctuidae		1900	1900	9	3	0	6	Forest	2	Egg
Gynaephora fascelina	Erebidae		1900	1900			1	6	Open	3	Larva
Hada plebeja	Noctuidae		1900	1900	4		1	6	Open	3	Pupa
Hadena albimacula	Noctuidae		1900	1900			0	4	Open	2	Pupa
Hadena confusa	Noctuidae		1900	1900			0	10	Open	2	Pupa
Hecatera bicolorata	Noctuidae		1900	1900			2	4	Open	3	Pupa
Helotropha leucostigma	Noctuidae		1900	1900	7		2	7	Generalist	3	Egg
Hemaris fuciformis	Sphingidae		1900	1900			0	8	Open	2	Pupa
Hemaris tityus	Sphingidae		1900	1900			0	11	Open	1	Pupa
Hepialus humuli	Hepialidae	NT	1900	1900	1		0	6	Open	3	Larva
Hoplodrina blanda	Noctuidae		1900	1900	36	17	0	7	Open	3	Larva
Hoplodrina octogenaria	Noctuidae		1900	1900	42		0	7	Generalist	3	Larva
Horisme tersata	Geometridae		1900	1900			0	7	Forest	3	Pupa
Hydraecia micacea	Noctuidae		1900	1900	38	32	1	10	Generalist	3	Egg
Hydrillula pallustris	Noctuidae		1900	1900			1	4	Open	2	Larva
Hydriomena furcata	Geometridae		1900	1900	19	2	2	7	Generalist	3	Egg
Hydriomena impluviata	Geometridae		1900	1900	6		2	7	Generalist	3	Pupa
Hydriomena ruberata	Geometridae		1900	1900			2	5	Generalist	2	Pupa
Hylaea fasciaria	Geometridae		1900	1900	17		2	7	Forest	2	Larva
Hyles gallii	Sphingidae		1900	1900			0	8	Generalist	2	Pupa
Hypena crassalis	Erebidae		1900	1900	91		2	6	Generalist	2	Pupa
Hypena proboscidalis	Erebidae		1900	1900	25		1	7	Generalist	2	Larva
Hypena rostralis	Erebidae		1900	1900			2	13	Generalist	1	Imago
Idaea aversata	Geometridae		1900	1900	103	123	1	8	Generalist	2	Larva
Idaea dimidiata	Geometridae		1900	1900			0	7	Generalist	2	Larva
Idaea emarginata	Geometridae		1900	1900		11	1	6	Forest	3	Larva
Idaea pallidata	Geometridae		1900	1900			0	5	Open	2	Larva
Idaea seriata	Geometridae		1900	1900			1	13	Generalist	2	Larva
Idaea serpentata	Geometridae		1900	1900			0	4	Open	2	Larva
Idaea straminata	Geometridae		1900	1900		4	0	6	Forest	3	Larva
Ipimorpha subtusa	Noctuidae		1900	1900			0	7	Forest	3	Egg
Jodis lactearia	Geometridae		1900	1900	7		0	3	Generalist	3	Pupa
Jodis putata	Geometridae		1900	1900			0	4	Generalist	2	Pupa
Lacanobia contigua	Noctuidae		1900	1900	1		0	9	Generalist	3	Pupa
Lacanobia oleracea	Noctuidae		1900	1900	6	1	0	13	Generalist	3	Pupa
Lacanobia suasa	Noctuidae		1900	1900			2	13	Generalist	3	Pupa
Laothoe populi	Sphingidae		1900	1900	65	1	1	11	Generalist	3	Pupa
Lasiocampa quercus	Lasiocampidae		1900	1900	2		1	5	Generalist	3	Pupa
Lasiocampa trifolii	Lasiocampidae		1900	1900		1	1	7	Generalist	3	Egg
Leucania comma	Noctuidae		1900	1900			0	9	Open	3	Larva
Leucoma salicis	Erebidae		1900	1900			0	7	Forest	3	Larva
Lithophane furcifera	Noctuidae		1900	1900			0	12	Forest	2	Imago
Lithophane ornitopus	Noctuidae		1900	1900			1	11	Forest	2	Imago
Lithosia quadra	Erebidae		1900	1900			0	6	Generalist	3	Larva
Lobophora halterata	Geometridae		1900	1900		5	1	5	Forest	3	Pupa
Lomaspilis marginata	Geometridae		1900	1900	26	2	2	10	Generalist	3	Pupa
Lycia hirtaria	Geometridae		1900	1900	87		1	9	Forest	3	Pupa
Lycophotia porphyrea	Noctuidae		1900	1900	50		0	7	Generalist	3	Larva
Lygephila craccae	Erebidae		1900	1900			0	7	Generalist	2	Egg
Lygephila pastinum	Erebidae		1900	1900	3	1	0	7	Generalist	2	Larva
Lygephila viciae	Erebidae		1900	1900			1	6	Forest	2	Pupa
Lymantria monacha	Erebidae		1900	1900	589		2	4	Generalist	2	Egg
Lythria cruentaria	Geometridae		1900	1900			2	8	Open	2	Pupa
Macaria brunneata	Geometridae		1900	1900	23		0	8	Generalist	3	Egg
Macaria liturata	Geometridae		1900	1900	57		0	10	Forest	2	Pupa
Macaria notata Macaria	Geometridae		1900	1900	3	1	0	7	Forest	3	Pupa
Macaria signaria	Geometridae		1900	1900	20		1	6	Forest	2	Pupa
Macaria wauaria	Geometridae		1900	1900	3	3	0	7	Forest	2	Egg
Macrothylacia rubi	Lasiocampidae		1900	1900			0	5	Generalist	3	Larva
Malacosoma castrensis	Lasiocampidae		1900	1900			0	7	Open	3	Egg
Malacosoma neustria	Lasiocampidae		1900	1900			1	6	Open	3	Egg
Mamestra brassicae	Noctuidae		1900	1900			1	15	Generalist	3	Pupa
Mesapamea secalis Mes	Noctuidae		1900	1900	42	30	2	6	Generalist	3	Larva
Mesoleuca albicillata	Geometridae		1900	1900	1		0	6	Generalist	2	Pupa
Mesotype didymata	Geometridae		1900	1900	1		1	7	Generalist	3	Egg
Mesotype parallelolinea	Geometridae		1900	1900			0	4	Open	2	Egg
Mimas tiliae	Sphingidae		1900	1900			2	8	Forest	2	Pupa
Mniotype adusta	Noctuidae		1900	1900	2		0	6	Open	3	Larva
Mythimna conigera	Noctuidae		1900	1900	23	26	0	7	Generalist	3	Larva
Mythimna ferrago	Noctuidae		1900	1900	45	8	0	7	Generalist	3	Larva
Mythimna pallens	Noctuidae		1900	1900			1	13	Generalist	3	Larva
Naenia typica	Noctuidae		1900	1900			0	8	Generalist	3	Larva
Noctua fimbriata	Noctuidae		1900	1900		15	2	8	Generalist	3	Larva
Noctua orbona	Noctuidae		1900	1900		5	1	11	Generalist	3	Larva
Noctua pronuba	Noctuidae		1900	1900	124	510	2	8	Generalist	3	Larva
Nola cucullatella	Nolidae		1900	1900			1	6	Forest	3	Larva
Notodonta dromedarius	Notodontidae		1900	1900	21	1	1	10	Forest	3	Pupa
Notodonta tritophus	Notodontidae		1900	1900			1	10	Forest	2	Pupa
Notodonta ziczac	Notodontidae		1900	1900	12	2	0	12	Generalist	3	Pupa
Nudaria mundana	Erebidae		1900	1900	33	2	0	5	Generalist	3	Larva
Nycteola revayana	Nolidae		1900	1900			2	13	Forest	2	Imago
Ochropacha duplaris	Drepanidae		1900	1900	12		1	9	Forest	3	Pupa
Ochropleura plecta	Noctuidae		1900	1900	18	1	0	13	Generalist	3	Larva
Odezia atrata	Geometridae		1900	1900		3	0	6	Open	3	Egg
Odontopera bidentata	Geometridae		1900	1900	28	1	2	5	Generalist	3	Pupa
Odontosia carmelita	Notodontidae		1900	1900	8		0	5	Generalist	3	Pupa

Oligia latruncula	Noctuidae		1900	1900		4	1	8	Generalist	3	Larva
Oligia strigilis	Noctuidae		1900	1900	9	4	1	6	Generalist	3	Larva
Operophtera brumata	Geometridae		1900	1900	39	33	0	4	Generalist	3	Egg
Opigena polygona	Noctuidae		1900	1900		1	0	7	Open	3	Larva
Opisthograptis luteolata	Geometridae		1900	1900		1	0	8	Generalist	3	Pupa
Orgyia antiqua	Erebidae		1900	1900	2		0	6	Generalist	3	Egg
Orthonama vittata	Geometridae		1900	1900			1	11	Generalist	2	Larva
Orthosia gothica	Noctuidae		1900	1900	1125	93	2	5	Generalist	3	Pupa
Orthosia incerta	Noctuidae		1900	1900	45	93	2	6	Generalist	3	Pupa
Panolis flammea	Noctuidae		1900	1900	51		0	5	Generalist	2	Pupa
Papestra biren	Noctuidae		1900	1900			0	5	Generalist	3	Pupa
Parascotia fuliginaria	Erebidae		1900	1900	3		0	6	Generalist	3	Larva
Parastichtis suspecta	Noctuidae		1900	1900	4		1	5	Forest	3	Egg
Parectropis similaria	Geometridae		1900	1900			1	6	Forest	2	Pupa
Pasiphila rectangulata	Geometridae		1900	1900	2		2	5	Forest	3	Egg
Pechipogo strigilata	Erebidae		1900	1900			1	6	Forest	3	Larva
Pelurga comitata	Geometridae		1900	1900			0	7	Generalist	3	Pupa
Perconia strigillaria	Geometridae		1900	1900			0	7	Open	2	Larva
Peribatodes secundaria	Geometridae		1900	1900	137		1	5	Forest	3	Larva
Peridea anceps	Notodontidae		1900	1900		1	1	6	Forest	2	Pupa
Perizoma affinitata	Geometridae		1900	1900			0	7	Generalist	1	Pupa
Perizoma albulata	Geometridae		1900	1900			1	10	Open	1	Pupa
Perizoma alchemillata	Geometridae		1900	1900	31		0	7	Generalist	2	Pupa
Perizoma flavofasciata	Geometridae	NT	1900	1900	1		0	7	Forest	1	Pupa
Petrophora chlorosata	Geometridae		1900	1900	3		0	4	Generalist	2	Pupa
Phalera bucephala	Notodontidae		1900	1900	50	2	0	8	Generalist	3	Pupa
Pheosia gnoma	Notodontidae		1900	1900	132	4	1	10	Generalist	2	Pupa
Pheosia tremula	Notodontidae		1900	1900			0	11	Generalist	2	Pupa
Phibalapteryx virgata	Geometridae		1900	1900			1	12	Open	2	Pupa
Philereme vetulata	Geometridae	NT	1900	1900		1	0	6	Open	2	Egg
Phragmatobia fuliginosa	Erebidae		1900	1900	4	2	0	3	Generalist	3	Larva
Phyllodesma ilicifolia	Lasiocampidae		1900	1900	3		0	6	Generalist	3	Pupa
Phymatopus hecta	Hepialidae		1900	1900	9		0	8	Generalist	3	Larva
Phytometra viridaria	Erebidae		1900	1900			0	9	Open	1	Pupa
Plagodis dolabraria	Geometridae		1900	1900	3		0	7	Forest	3	Pupa
Plagodis pulveraria	Geometridae		1900	1900	1		1	5	Generalist	3	Pupa
Plemyria rubiginata	Geometridae		1900	1900	2		0	5	Forest	3	Egg
Plusia festucae Plusia pu	Noctuidae		1900	1900		1	0	4	Generalist	3	Larva
Poecilocampa populi	Lasiocampidae		1900	1900	38	2	0	3	Generalist	3	Egg
Polia bombycina	Noctuidae		1900	1900	3		1	7	Generalist	3	Larva
Polia hepatica	Noctuidae		1900	1900	2		0	7	Generalist	3	Larva
Polia nebulosa	Noctuidae		1900	1900	8		0	4	Generalist	3	Larva
Polymixis polymita	Noctuidae		1900	1900			0	6	Open	3	Egg
Polypogon tentacularia	Erebidae		1900	1900			0	8	Generalist	3	Larva
Pseudoips prasinana	Nolidae		1900	1900			0	7	Forest	3	Pupa
Pterapherapteryx sexalata	Geometridae		1900	1900			0	6	Generalist	3	Pupa
Pterostoma palpina	Notodontidae		1900	1900	10	1	1	11	Generalist	3	Pupa
Ptilodon capucina	Notodontidae		1900	1900	29		1	11	Forest	3	Pupa
Ptilophora plumigera	Notodontidae		1900	1900		8	1	2	Forest	1	Egg
Rheumaptera hastata	Geometridae		1900	1900			1	5	Open	3	Pupa
Rheumaptera undulata	Geometridae		1900	1900	5		0	7	Generalist	3	Pupa
Rhodostrophia vibicaria	Geometridae		1900	1900			2	8	Open	3	Larva
Rhyacia simulans	Noctuidae		1900	1900			0	8	Generalist	3	Larva
Rivula sericealis	Erebidae		1900	1900			1	11	Generalist	3	Larva
Rusina ferruginea	Noctuidae		1900	1900	111	43	0	6	Generalist	3	Larva
Saturnia pavonia	Saturniidae		1900	1900			0	8	Generalist	3	Pupa
Scoliopteryx libatrix	Erebidae		1900	1900	1		0	18	Generalist	3	Imago
Scopula floslactata	Geometridae		1900	1900			1	4	Forest	3	Larva
Scopula immorata	Geometridae		1900	1900			0	6	Open	2	Larva
Scopula immutata	Geometridae		1900	1900	27		0	7	Open	3	Larva
Scopula incanata	Geometridae		1900	1900	1		1	6	Open	3	Larva
Scopula ternata	Geometridae		1900	1900	3		0	6	Open	3	Larva
Scotopteryx chenopodiata	Geometridae		1900	1900		10	1	6	Generalist	2	Larva
Selenia dentaria	Geometridae		1900	1900			1	12	Generalist	3	Pupa
Selenia lunularia	Geometridae		1900	1900			1	7	Forest	3	Pupa
Setina irrorella	Erebidae		1900	1900			1	6	Open	3	Larva
Sideridis reticulata	Noctuidae		1900	1900			0	7	Open	3	Pupa
Sideridis rivularis	Noctuidae		1900	1900	11	2	0	12	Open	2	Pupa
Siona lineata	Geometridae		1900	1900	6		0	7	Open	3	Larva
Smerinthus ocellata	Sphingidae		1900	1900	15	2	0	8	Forest	3	Pupa
Spaelotis ravida	Noctuidae		1900	1900		3	0	9	Open	3	Larva
Sphinx ligustri	Sphingidae		1900	1900			0	6	Generalist	2	Pupa
Sphinx pinastri	Sphingidae		1900	1900	58		0	13	Forest	2	Pupa
Spilosoma lubricipeda	Erebidae		1900	1900	62		2	3	Open	3	Pupa
Standfussiana lucernea	Noctuidae		1900	1900			0	8	Generalist	2	Larva
Staurophora celsia	Noctuidae		1900	1900			0	4	Open	3	Egg
Stauropus fagi	Notodontidae		1900	1900	2		0	11	Forest	3	Pupa
Syngrapha interrogationis	Noctuidae		1900	1900			0	6	Generalist	3	Larva
Tethea or	Drepanidae		1900	1900	8	1	2	7	Forest	3	Pupa
Thalera fimbrialis	Geometridae		1900	1900			0	7	Generalist	2	Larva
Thera cognata	Geometridae		1900	1900	3		1	6	Open	2	Egg
Thera juniperata	Geometridae		1900	1900	11		1	4	Open	2	Egg
Thera variata	Geometridae		1900	1900	74	4	2	5	Forest	2	Larva
Tholera cespitis	Noctuidae		1900	1900		13	0	5	Open	3	Egg
Tholera decimalis	Noctuidae		1900	1900	30	5	0	5	Open	3	Egg
Thyatira batis	Drepanidae		1900	1900	2		0	10	Generalist	3	Pupa
Tiliacea citrago	Noctuidae		1900	1900	3		1	4	Forest	1	Egg
Timandra comae Timan	Geometridae		1900	1900	22	22	0	14	Generalist	3	Larva
Trichopteryx carpinata	Geometridae		1900	1900			0	5	Generalist	3	Pupa
Triodia sylvina	Hepialidae		1900	1900	14	72	1	4	Generalist	3	Larva
Triphosa dubitata	Geometridae		1900	1900			0	14	Open	3	Larva
Xanthia togata	Noctuidae		1900	1900	4	9	0	7	Generalist	3	Egg
Xanthorhoe designata	Geometridae		1900	1900	3		0	10	Forest	1	Pupa
Xanthorhoe ferrugata	Geometridae		1900	1900	104	13	2	12	Generalist	3	Pupa
Xanthorhoe fluctuata	Geometridae		1900	1900	4	28	1	13	Generalist	3	Pupa
Xanthorhoe montanata	Geometridae		1900	1900	33		2	6	Generalist	3	Larva
Xanthorhoe quadrifasiata	Geometridae		1900	1900	4		1	7	Generalist	3	Larva
Xanthorhoe spadicearia	Geometridae		1900	1900	7		1	11	Forest	3	Pupa
Xestia c-nigrum	Noctuidae		1900	1900	19	102	1	16	Generalist	3	Larva
Xestia triangulum	Noctuidae		1900	1900	10	15	0	7	Generalist	3	Larva
Xylena exsoleta	Noctuidae		1900	1900			0	11	Open	3	Imago
Xylena vetusta	Noctuidae		1900	1900		1	0	12	Generalist	3	Imago
Acherontia atropos	Sphingidae		1935	1900			1	10	Generalist	2	Pupa
Acronicta alni	Noctuidae		1935	1900			2	6	Forest	3	Pupa
Agrius convolvuli	Sphingidae		1935	1900			0	9	Generalist	2	Pupa
Apamea sublustris	Noctuidae		1935	1900			0	5	Open	3	Larva
Biston strataria	Geometridae		1935	1900	20	26	1	5	Forest	3	Pupa
Catocala pacta	Erebidae		1935	1900			0	6	Generalist	2	Egg
Chersotis cuprea	Noctuidae		1935	1900			0	6	Open	3	Larva
Coenophila subrosea	Noctuidae		1935	1900	4		0	4	Generalist	3	Larva
Craniophora ligustri	Noctuidae	NT	1935	1900	7		1	9	Forest	3	Pupa
Deileptenia ribeata	Geometridae		1935	1900	181		1	6	Forest	3	Larva
Drepana curvatula	Drepanidae		1935	1900			1	9	Forest	3	Pupa
Elophos vittaria	Geometridae		1935	1900			0	5	Open	3	Larva
Erannis defoliaria	Geometridae		1935	1900	8	5	2	3	Generalist	3	Egg
Eupithecia conterminata	Geometridae		1935	1900			0	5	Forest	2	Pupa
Hemistola chrysoprasaria	Geometridae		1935	1900			0	6	Open	1	Larva
Idaea biselata	Geometridae		1935	1900	17	5	1	6	Generalist	2	Larva
Korscheltellus fusconebu	Hepialidae		1935	1900	26	1	1	7	Generalist	3	Larva
Larentia clavaria	Geometridae		1935	1900	1		1	3	Open	2	Egg
Lasionhada proxima	Noctuidae		1935	1900			0	9	Generalist	3	Larva
Laspeyria flexula	Erebidae		1935	1900	3		0	6	Generalist	3	Larva
Macaria carbonaria	Geometridae		1935	1900			0	6	Generalist	2	Pupa
Nonagria typhae	Noctuidae		1935	1900	2	3	2	9	Generalist	1	Egg
Perizoma blandiata	Geometridae		1935	1900	1		1	4	Open	1	Pupa
Phigalia pilosaria	Geometridae		1935	1900			1	3	Generalist	3	Pupa

Photedes captiuncula	Noctuidae		1935	1900			0	6	Open	2	Larva
Thalpophila matura	Noctuidae		1935	1900			1	4	Open	3	Larva
Xanthorhoe biriviata	Geometridae		1935	1900			1	12	Forest	1	Pupa
Xestia baja	Noctuidae		1935	1900	40	2	0	6	Generalist	3	Larva
Xestia xanthographa	Noctuidae		1935	1900	139	704	2	6	Generalist	3	Larva
Epilecta linogrisea	Noctuidae		1971	1900			0	5	Generalist	3	Larva
Cosmia pyralina	Noctuidae		1978	1900			0	5	Forest	2	Egg
Ecliptopera capitata	Geometridae	VU	2003	1900	1		0	5	Forest	1	Pupa
Xylocampa areola	Noctuidae		2019	1900			0	6	Open	1	Pupa
Epirrhoe hastulata	Geometridae			1900			1	4	Open	2	Pupa
Eriopygodes imbecilla	Noctuidae			1900			0	5	Open	1	Larva
Aglia tau	Saturniidae		1900	1935			0	5	Forest	2	Pupa
Agrochola nitida	Noctuidae		1900	1935	4	22	0	5	Forest	3	Egg
Calliteara pudibunda	Erebidae		1900	1935	6		2	6	Forest	3	Larva
Denticucullus pygmina	Noctuidae		1900	1935	41		1	6	Open	2	Egg
Diarsia dahlii	Noctuidae		1900	1935	19		1	6	Forest	3	Larva
Eilema complana	Erebidae		1900	1935	85	30	0	9	Generalist	3	Larva
Ennomos alniaria	Geometridae		1900	1935	56		1	9	Generalist	3	Egg
Eupithecia lariciata	Geometridae		1900	1935			1	5	Forest	2	Pupa
Furcula bicuspis	Notodontidae		1900	1935			0	5	Forest	3	Pupa
Furcula bifida	Notodontidae		1900	1935	1		0	8	Forest	3	Pupa
Herminia tarsipennalis	Erebidae		1900	1935	3	3	1	7	Generalist	3	Larva
Leucania obsoleta	Noctuidae		1900	1935			0	12	Generalist	3	Larva
Leucodonta bicoloria	Notodontidae		1900	1935	6		0	8	Forest	2	Pupa
Ligdia adustata	Geometridae		1900	1935	2	1	0	11	Forest	1	Pupa
Lithophane lamda	Noctuidae		1900	1935			0	12	Generalist	2	Imago
Lithophane socia	Noctuidae		1900	1935	11	1	0	12	Forest	3	Imago
Macroglossum stellatar	Sphingidae		1900	1935			1	7	Generalist	2	Imago
Meganola strigula	Nolidae		1900	1935			0	4	Forest	2	Larva
Noctua comes	Noctuidae		1900	1935	66	86	1	8	Generalist	3	Larva
Nola confusalis	Nolidae		1900	1935			0	6	Forest	3	Pupa
Notodonta torva	Notodontidae		1900	1935			0	10	Forest	2	Pupa
Nycteola degenerana	Nolidae		1900	1935			1	13	Forest	3	Imago
Orgyia recens	Erebidae		1900	1935			0	2	Generalist	3	Larva
Panthea coenobita	Noctuidae		1900	1935	9		0	7	Forest	2	Pupa
Pasiphila debiliata	Geometridae		1900	1935			0	6	Generalist	2	Egg
Schrankia costaestrigali	Erebidae		1900	1935			1	5	Generalist	3	Larva
Sphrageidus similis	Erebidae		1900	1935			0	5	Forest	3	Larva
Spilarctia lutea	Erebidae		1900	1935	12		0	7	Open	3	Pupa
Thumatha senex	Erebidae		1900	1935			0	8	Generalist	2	Larva
Xestia stigmatica	Noctuidae		1900	1935		7	0	5	Generalist	3	Larva
Xylena solidaginis	Noctuidae		1900	1935	14		1	8	Generalist	3	Egg
Abraxas sylvata	Geometridae		1935	1935			0	6	Forest	1	Pupa
Acasis viretata	Geometridae		1935	1935			1	4	Forest	2	Pupa
Achlya flavicornis	Drepanidae		1935	1935	20		1	3	Forest	3	Pupa
Actebia praecox	Noctuidae		1935	1935			0	7	Open	3	Larva
Aethalura punctulata	Geometridae		1935	1935			1	5	Forest	2	Pupa
Agriopis aurantiaria	Geometridae		1935	1935	17		0	3	Forest	3	Egg
Agriopis leucophaearia	Geometridae		1935	1935		3	2	3	Forest	2	Pupa
Agrochola macilenta	Noctuidae		1935	1935	3	16	1	2	Forest	3	Egg
Agrotis cinerea	Noctuidae		1935	1935			2	7	Open	3	Larva
Amphipoea crinanensis	Noctuidae	NT	1935	1935		1	1	7	Generalist	1	Egg
Amphipoea fucosa	Noctuidae		1935	1935	2	36	1	8	Generalist	3	Egg
Amphipoea lucens	Noctuidae		1935	1935			1	7	Generalist	3	Egg
Amphipyra tragopoginis	Noctuidae		1935	1935		9	0	9	Generalist	3	Egg
Anticlea derivata	Geometridae		1935	1935			0	6	Forest	1	Pupa
Anticollix sparsata	Geometridae		1935	1935			0	4	Generalist	2	Pupa
Apamea anceps	Noctuidae		1935	1935			0	5	Open	3	Larva
Apamea illyria	Noctuidae		1935	1935	2		0	4	Forest	3	Larva
Apamea lithoxylaea	Noctuidae		1935	1935			0	6	Open	3	Larva
Apamea remissa	Noctuidae		1935	1935	3	2	2	8	Generalist	3	Larva
Apamea unanimis	Noctuidae		1935	1935	4		0	6	Open	3	Larva
Apeira syringaria	Geometridae		1935	1935			0	4	Forest	2	Larva
Aporophyla lueneburge	Noctuidae		1935	1935		19	2	4	Open	3	Egg
Archanara dissoluta	Noctuidae		1935	1935		1	1	7	Generalist	1	Egg
Archiearis notha	Geometridae		1935	1935			1	6	Forest	2	Pupa
Arctornis l-nigrum	Erebidae		1935	1935			0	16	Forest	3	Larva
Arenostola phragmitidis	Noctuidae		1935	1935		1	0	5	Generalist	1	Egg
Autographa bractea	Noctuidae		1935	1935			0	6	Open	3	Larva
Bena bicolorana	Nolidae		1935	1935			0	7	Forest	2	Larva
Brachionycha nubeculos	Noctuidae		1935	1935			0	6	Forest	3	Pupa
Bryophila raptricula	Noctuidae		1935	1935			1	7	Generalist	3	Larva
Calamia tridens	Noctuidae		1935	1935			0	6	Open	2	Egg
Calophasia lunula	Noctuidae		1935	1935		47	0	10	Generalist	1	Pupa
Caradrina montana	Noctuidae		1935	1935			0	5	Forest	3	Larva
Caradrina selini	Noctuidae		1935	1935			0	6	Forest	3	Larva
Catocala promissa	Erebidae		1935	1935			0	4	Forest	2	Egg
Catocala sponsa	Erebidae		1935	1935	2		0	6	Forest	2	Egg
Celaena haworthii	Noctuidae		1935	1935	2		0	6	Generalist	3	Egg
Cepphis advenaria	Geometridae		1935	1935	7		0	7	Generalist	2	Pupa
Chilodes maritima	Noctuidae		1935	1935	1	1	2	4	Generalist	2	Larva
Chloantha hyperici	Noctuidae		1935	1935			0	10	Generalist	2	Pupa
Cilix glaucata	Drepanidae		1935	1935			0	9	Generalist	2	Pupa
Cirrhia gilvago	Noctuidae		1935	1935			1	6	Open	1	Egg
Colostygia olivata	Geometridae		1935	1935	2		0	5	Open	2	Larva
Conistra erythrocephala	Noctuidae		1935	1935		20	2	11	Forest	3	Imago
Conistra rubiginosa	Noctuidae		1935	1935		7	0	10	Forest	3	Imago
Coranarta cordigera	Noctuidae		1935	1935			0	4	Generalist	2	Pupa
Cosmotriche lobulina	Lasiocampidae		1935	1935			0	6	Forest	2	Larva
Crypsedra gemmea	Noctuidae		1935	1935	7		0	6	Open	3	Egg
Cucullia absinthii	Noctuidae		1935	1935			0	6	Generalist	2	Pupa
Cucullia chamomillae	Noctuidae		1935	1935			0	4	Open	2	Pupa
Cucullia lactucae	Noctuidae		1935	1935			0	8	Forest	2	Pupa
Cucullia lucifuga	Noctuidae		1935	1935			0	6	Forest	2	Pupa
Cucullia umbratica	Noctuidae		1935	1935	2		0	6	Generalist	3	Pupa
Cyclophora linearia	Geometridae		1935	1935			1	7	Forest	1	Pupa
Cyclophora quercimonta	Geometridae		1935	1935			0	6	Forest	2	Pupa
Deltote pygarga	Noctuidae		1935	1935	264	1	0	6	Forest	2	Pupa
Dyscia fagaria	Geometridae		1935	1935			0	5	Open	2	Pupa
Dysstroma infuscata	Geometridae		1935	1935	3		1	3	Generalist	2	Larva
Dysstroma latefasciata	Geometridae		1935	1935	81	2	2	7	Generalist	2	Larva
Earophila badiata	Geometridae		1935	1935			1	5	Open	1	Pupa
Eilema depressa	Erebidae		1935	1935	11174	1	0	12	Generalist	3	Larva
Ennomos fuscantaria	Geometridae		1935	1935		1	1	9	Forest	1	Egg
Ennomos quercinaria	Geometridae		1935	1935	1		1	7	Forest	2	Egg
Epipsilia grisescens	Noctuidae		1935	1935			0	4	Generalist	3	Egg
Epirrhoe rivata	Geometridae		1935	1935			0	7	Open	2	Pupa
Epirrita christyi	Geometridae		1935	1935			1	3	Forest	3	Egg
Epirrita dilutata	Geometridae		1935	1935			1	3	Forest	3	Egg
Eugnorisma glareosa	Noctuidae		1935	1935	13		1	5	Open	2	Larva
Eugraphe sigma	Noctuidae		1935	1935			0	6	Open	3	Larva
Eulithis mellinata	Geometridae		1935	1935	13		1	8	Forest	2	Egg
Euphyia unangulata	Geometridae		1935	1935	5		0	10	Forest	2	Pupa
Eupithecia actaeata	Geometridae		1935	1935			1	8	Forest	1	Pupa
Eupithecia analoga	Geometridae		1935	1935			0	4	Forest	2	Pupa
Eupithecia assimilata	Geometridae		1935	1935			0	9	Generalist	1	Pupa
Eupithecia denotata	Geometridae		1935	1935			1	6	Open	2	Pupa
Eupithecia dodoneata	Geometridae		1935	1935			1	6	Forest	2	Pupa
Eupithecia exiguata	Geometridae		1935	1935		5	0	5	Generalist	2	Pupa
Eupithecia gelidata	Geometridae		1935	1935			0	6	Generalist	1	Pupa
Eupithecia goossensiata	Geometridae		1935	1935			0	4	Generalist	2	Pupa
Eupithecia indigata	Geometridae		1935	1935	1		0	5	Forest	2	Pupa
Eupithecia innotata	Geometridae		1935	1935			1	12	Open	2	Pupa
Eupithecia insigniata	Geometridae		1935	1935			0	5	Forest	2	Pupa
Eupithecia inturbata	Geometridae		1935	1935			0	4	Forest	1	Egg
Eupithecia irriguata	Geometridae		1935	1935		23	0	7	Forest	2	Pupa

Eupithecia linariata	Geometridae		1935	1935			0	10	Open	1	Pupa
Eupithecia nanata	Geometridae		1935	1935			1	11	Open	2	Pupa
Eupithecia pimpinellata	Geometridae		1935	1935			1	6	Open	2	Pupa
Eupithecia plumbeolata	Geometridae		1935	1935			0	7	Forest	2	Pupa
Eupithecia pygmaeata	Geometridae		1935	1935			1	4	Open	2	Pupa
Eupithecia sinuosaria	Geometridae		1935	1935			0	7	Generalist	2	Pupa
Eupithecia subumbrata	Geometridae	NT	1935	1935	1		1	6	Open	3	Pupa
Eupithecia tantillaria	Geometridae		1935	1935			0	5	Forest	3	Pupa
Eupithecia tenuiata	Geometridae		1935	1935			1	6	Forest	2	Egg
Eupithecia tripunctaria	Geometridae		1935	1935			1	11	Open	2	Pupa
Eupithecia valerianata	Geometridae		1935	1935			0	6	Open	2	Pupa
Eupithecia venosata	Geometridae		1935	1935			0	5	Open	2	Pupa
Eupithecia virgaureata	Geometridae		1935	1935			1	4	Generalist	3	Pupa
Eustroma reticulata	Geometridae		1935	1935			0	7	Forest	1	Pupa
Fagivorina arenaria	Geometridae		1935	1935			0	5	Forest	2	Pupa
Fissipunctia ypsillon	Noctuidae		1935	1935	1		1	5	Forest	2	Egg
Globia algae	Noctuidae		1935	1935			0	4	Generalist	1	Egg
Globia sparganii	Noctuidae		1935	1935			0	6	Generalist	1	Egg
Gortyna flavago	Noctuidae		1935	1935	27		0	8	Generalist	2	Egg
Gymnoscelis rufifasciata	Geometridae		1935	1935			1	15	Generalist	3	Pupa
Hadena perplexa	Noctuidae	VU	1935	1935		1	0	7	Open	2	Pupa
Hemithea aestivaria	Geometridae		1935	1935			0	7	Forest	3	Larva
Herminia grisealis	Erebidae		1935	1935	6		0	6	Forest	3	Pupa
Hydrelia flammeolaria	Geometridae		1935	1935	9		0	6	Forest	2	Pupa
Hyles livornica	Sphingidae		1935	1935			0	12	Generalist	3	Egg
Hypenodes humidalis	Erebidae		1935	1935			0	5	Generalist	3	Larva
Hypomecis roboraria	Geometridae		1935	1935	7		1	4	Forest	3	Larva
Hyppa rectilinea	Noctuidae		1935	1935	5		1	6	Forest	3	Larva
Idaea deversaria	Geometridae		1935	1935			0	4	Open	2	Larva
Idaea muricata	Geometridae		1935	1935			1	4	Generalist	3	Larva
Korscheltellus lupulina	Hepialidae		1935	1935	6	41	0	5	Generalist	3	Larva
Lacanobia thalassina	Noctuidae		1935	1935	18		0	7	Generalist	3	Pupa
Lampropteryx otregiata	Geometridae		1935	1935			0	6	Forest	1	Pupa
Lateroligia ophiogramm	Noctuidae		1935	1935	5		2	6	Forest	3	Larva
Lithophane consocia	Noctuidae		1935	1935			0	11	Generalist	2	Imago
Litoligia literosa	Noctuidae		1935	1935			1	5	Open	2	Larva
Lomographa bimaculata	Geometridae		1935	1935	2		0	8	Forest	3	Pupa
Lomographa temerata	Geometridae		1935	1935	8	1	1	7	Forest	3	Pupa
Luperina testacea	Noctuidae		1935	1935	7	2269	1	6	Generalist	3	Larva
Lymantria dispar	Erebidae		1935	1935		1	0	4	Generalist	2	Egg
Macdunnoughia confusa	Noctuidae		1935	1935		1	0	14	Generalist	3	Larva
Macrochilo cribrumalis	Erebidae		1935	1935			0	5	Generalist	3	Larva
Malacodea regelaria	Geometridae		1935	1935			0	6	Forest	2	Pupa
Martania taeniata	Geometridae		1935	1935	2		1	6	Generalist	3	Larva
Melanchra persicariae	Noctuidae		1935	1935			1	8	Generalist	3	Pupa
Mesoligia furuncula	Noctuidae		1935	1935		47	1	5	Generalist	3	Larva
Mniotype satura	Noctuidae		1935	1935	55		0	5	Forest	3	Egg
Mythimna impura	Noctuidae		1935	1935	22	30	0	7	Generalist	3	Larva
Mythimna pudorina	Noctuidae		1935	1935			0	7	Open	3	Larva
Mythimna turca	Noctuidae		1935	1935			0	5	Forest	3	Larva
Odontosia sieversii	Notodontidae		1935	1935			1	6	Forest	2	Pupa
Oligia fasciuncula	Noctuidae		1935	1935			0	5	Open	3	Larva
Operophtera fagata	Geometridae		1935	1935	28	10	0	4	Forest	3	Egg
Orgyia antiquoides	Erebidae		1935	1935			0	9	Open	1	Egg
Orthosia cerasi	Noctuidae		1935	1935	30	265	0	6	Generalist	3	Pupa
Orthosia cruda	Noctuidae		1935	1935	18	521	1	6	Forest	3	Pupa
Orthosia gracilis	Noctuidae		1935	1935		1	0	4	Generalist	3	Pupa
Orthosia miniosa	Noctuidae		1935	1935	15	8	1	4	Forest	2	Pupa
Orthosia opima	Noctuidae		1935	1935	21		0	5	Forest	3	Pupa
Orthosia populeti	Noctuidae		1935	1935	21	13	1	6	Forest	2	Pupa
Ourapteryx sambucaria	Geometridae		1935	1935			0	7	Generalist	3	Larva
Paradarisa consonaria	Geometridae		1935	1935			2	5	Forest	3	Pupa
Pasiphila chloerata	Geometridae		1935	1935			1	5	Forest	2	Egg
Pennithera firmata	Geometridae		1935	1935	113		0	6	Forest	2	Egg
Perizoma bifaciata	Geometridae		1935	1935			0	5	Open	1	Pupa
Perizoma hydrata	Geometridae		1935	1935			0	6	Open	1	Pupa
Phlogophora meticulosa	Noctuidae		1935	1935	8	2	1	8	Generalist	3	Egg
Photedes fluxa	Noctuidae		1935	1935		5	1	4	Generalist	3	Larva
Photedes minima	Noctuidae		1935	1935	6	1	0	7	Generalist	2	Larva
Polychrysia moneta	Noctuidae		1935	1935			0	7	Open	2	Larva
Protolampra sobrina	Noctuidae		1935	1935	1		0	4	Forest	3	Larva
Pyrrhia umbra	Noctuidae		1935	1935			0	8	Generalist	3	Pupa
Rheumaptera cervinalis	Geometridae		1935	1935			0	6	Generalist	1	Pupa
Rhizedra lutosa	Noctuidae		1935	1935	2	23	1	6	Generalist	3	Egg
Scopula rubiginata	Geometridae		1935	1935			1	10	Open	1	Larva
Selenia tetralunaria	Geometridae		1935	1935	7		1	11	Forest	3	Pupa
Spargania luctuata	Geometridae		1935	1935			1	11	Generalist	2	Pupa
Spilosoma urticae	Erebidae		1935	1935			0	8	Generalist	3	Larva
Syngrapha microgamma	Noctuidae		1935	1935			0	4	Generalist	3	Larva
Tetheella fluctuosa	Drepanidae		1935	1935	59		2	5	Forest	2	Pupa
Thera obeliscata	Geometridae		1935	1935	160	10	2	16	Forest	2	Larva
Tiliacea aurago	Noctuidae		1935	1935	11	5	1	5	Forest	2	Egg
Trachea atriplicis	Noctuidae		1935	1935	1	1	0	8	Generalist	3	Pupa
Trichiura crataegi	Lasiocampidae		1935	1935	10		0	5	Generalist	3	Egg
Trisateles emortualis	Erebidae		1935	1935			0	8	Forest	2	Pupa
Venusia cambrica	Geometridae		1935	1935			0	7	Forest	2	Pupa
Xanthorhoe decoloraria	Geometridae		1935	1935			1	5	Open	2	Larva
Xestia alpicola	Noctuidae		1935	1935			0	4	Forest	3	Larva
Xestia ashworthii	Noctuidae		1935	1935			2	8	Open	3	Larva
Xestia castanea	Noctuidae		1935	1935			1	6	Open	3	Larva
Xestia sexstrigata	Noctuidae		1935	1935	73	35	0	4	Forest	3	Larva
Xestia sincera	Noctuidae		1935	1935			0	5	Forest	2	Larva
Xestia speciosa	Noctuidae		1935	1935			1	7	Forest	3	Larva
Zeuzera pyrina	Cossidae		1935	1935			0	6	Generalist	3	Larva
Ipimorpha retusa	Noctuidae		1969	1935			0	7	Forest	2	Egg
Lampropteryx suffumata	Geometridae		1972	1935	1	2	0	7	Open	2	Pupa
Cucullia asteris	Noctuidae		1974	1935			0	7	Open	2	Pupa
Polyploca ridens	Drepanidae		1978	1935			1	4	Forest	2	Pupa
Cucullia gnaphalii	Noctuidae		1984	1935			0	5	Open	2	Pupa
Euxoa cursoria	Noctuidae		1989	1935			2	8	Open	3	Larva
Cymatophorina diluta	Drepanidae		2003	1935			1	4	Forest	2	Egg
Daphnis nerii	Sphingidae		2011	1935			0	11	Open	1	Pupa
Longalatedes elymi	Noctuidae		2011	1935			0	6	Generalist	1	Larva
Rheumaptera subhastata	Geometridae		2011	1935			1	5	Generalist	3	Pupa
Agrotis ripae	Noctuidae			1935			1	6	Generalist	3	Larva
Eupithecia cauchiata	Geometridae			1935			1	6	Forest	2	Pupa
Eupithecia immundata	Geometridae			1935			0	3	Forest	1	Pupa
Euxoa recussa	Noctuidae			1935			1	5	Open	3	Larva
Hadena caesia	Noctuidae			1935			0	6	Generalist	1	Larva
Mesogona oxalina	Noctuidae			1935			0	4	Generalist	2	Egg
Mythimna straminea	Noctuidae			1935			0	7	Generalist	3	Larva
Paracolax tristalis	Erebidae			1935			0	7	Forest	3	Larva
Polymixis flavicincta	Noctuidae			1935			0	4	Generalist	2	Egg
Scotopteryx mucronata	Geometridae			1935			1	4	Open	1	Pupa
Trichopteryx polycommata	Geometridae			1935			0	4	Forest	1	Pupa
Zanclognatha lunalis	Erebidae			1935			1	6	Generalist	3	Larva
Hydraecia ultima	Noctuidae		1935	1945		1	1	4	Generalist	1	Egg
Nola aerugula	Nolidae		1935	1957			2	6	Generalist	3	Larva
Amphipyra perflua	Noctuidae		1935	1967			0	4	Forest	2	Egg
Ennomos autumnaria	Geometridae		1900	1968			0	7	Forest	3	Egg
Asteroscopus sphinx	Noctuidae		1984	1968			0	3	Forest	3	Egg
Gluphisia crenata	Notodontidae		1935	1969	1		1	7	Forest	3	Pupa
Eilema cereola	Erebidae		1935	1971			0	5	Generalist	3	Larva
Euthrix potatoria	Lasiocampidae		1976	1971			1	7	Generalist	3	Larva
Eupithecia egenaria	Geometridae		1935	1974			0	3	Forest	1	Pupa
Hydraecia petasitis	Noctuidae		2010	1974			0	5	Generalist	1	Egg

Pachycnemia hippocast	Geometridae			1975			0	13	Open	2	Pupa
Drymonia dodonaea	Notodontidae		1935	1976			0	8	Forest	3	Pupa
Eremobia ochroleuca	Noctuidae		1935	1976		1	0	7	Open	3	Larva
Peribatodes rhomboida	Geometridae		1986	1976	1	3	1	6	Generalist	3	Larva
Eugnorisma depuncta	Noctuidae		1935	1977		1	0	4	Forest	3	Larva
Apamea scolopacina	Noctuidae		1935	1978	14		1	6	Forest	3	Larva
Gagitodes sagittata	Geometridae		1978	1978			0	5	Forest	1	Pupa
Heterothera serraria	Geometridae		1985	1978			0	5	Forest	2	Larva
Eupithecia trisignaria	Geometridae		1935	1979			1	9	Open	2	Pupa
Watsonalla cultraria	Drepanidae		1935	1983			1	9	Forest	2	Pupa
Idaea sylvestraria	Geometridae		1900	1984			0	6	Open	2	Larva
Chloroclystis v-ata	Geometridae		1993	1984		1	0	10	Forest	3	Pupa
Actebia fennica	Noctuidae			1984			1	7	Open	3	Larva
Apamea rubrirena	Noctuidae		1935	1985	5		0	6	Forest	3	Larva
Habrosyne pyritoides	Drepanidae		1935	1985	1		0	8	Generalist	3	Pupa
Clostera anastomosis	Notodontidae		1900	1989			1	5	Forest	2	Larva
Philereme transversata	Geometridae		1935	1989			0	6	Open	3	Egg
Noctua janthe	Noctuidae		1992	1991	4	9	1	7	Generalist	3	Larva
Venusia blomeri	Geometridae		1985	1993			0	5	Forest	1	Pupa
Tethea ocularis	Drepanidae		1935	1994			0	3	Forest	2	Pupa
Pseudopanthera macula	Geometridae		1935	1995			0	5	Generalist	3	Pupa
Chesias legatella	Geometridae		1935	1996			0	4	Generalist	1	Egg
Nycteola asiatica	Nolidae		1935	1998			0	7	Generalist	3	Imago
Protoschinia scutosa	Noctuidae		1999	1998			0	7	Generalist	2	Pupa
Orthonama obstipata	Geometridae		2016	2001			2	11	Generalist	2	Imago
Eucarta virgo	Noctuidae		2004	2002			0	12	Generalist	2	Pupa
Autographa macrogami	Noctuidae			2003			0	6	Open	3	Larva
Chrysodeixis chalcites	Noctuidae			2004			0	16	Generalist	3	Larva
Autographa mandarina	Noctuidae		1935	2005			0	11	Generalist	3	Larva
Peridroma saucia	Noctuidae		2003	2006			2	9	Generalist	3	Larva
Hadena bicruris Hadena	Noctuidae	NT	1900	2010		1	0	13	Generalist	2	Pupa
Apocheima hispidaria	Geometridae		1935	2010	2	4	1	6	Forest	2	Pupa
Comibaena bajularia	Geometridae		1935	2010			0	4	Forest	2	Larva
Eupithecia abbreviata	Geometridae		1935	2010			1	7	Forest	2	Pupa
Lenisa geminipuncta	Noctuidae		1935	2010			1	6	Generalist	1	Egg
Noctua interjecta	Noctuidae		1935	2010	2	3	1	4	Generalist	3	Larva
Tyria jacobaeae	Erebidae		1935	2010			0	3	Open	1	Pupa
Miltochrista miniata	Erebidae		1989	2010	196		1	5	Open	3	Larva
Anorthoa munda	Noctuidae		1991	2010	14	7	0	5	Forest	3	Pupa
Watsonalla binaria	Drepanidae		1995	2010	3	16	1	10	Forest	2	Pupa
Moma alpium	Noctuidae		1935	2011	1		0	4	Forest	3	Pupa
Eilema sororcula	Erebidae		1986	2011		9	0	3	Forest	3	Pupa
Thera britannica	Geometridae			2011			2	11	Generalist	1	Egg
Eupithecia millefoliata	Geometridae		1935	2013			1	7	Open	2	Pupa
Bryophila domestica	Noctuidae		1935	2014			0	5	Generalist	3	Larva
Hypomecis punctinalis	Geometridae		1935	2014	19	3	1	7	Forest	3	Pupa
Aplocera efformata	Geometridae		1977	2014			0	12	Open	2	Larva
Hoplodrina ambigua	Noctuidae		2009	2014		337	0	6	Open	3	Larva
Nyctobrya muralis	Noctuidae			2015			0	5	Generalist	3	Larva
Idaea fuscovenosa	Geometridae		1900	2016			0	4	Open	2	Larva
Helicoverpa armigera	Noctuidae		2006	2016			0	13	Generalist	3	Pupa
Clostera anachoreta	Notodontidae		1900	2017			0	5	Forest	2	Pupa
Eupithecia haworthiata	Geometridae		1982	2019			0	6	Open	1	Pupa
Eupithecia ochridata	Geometridae		1989	2019			1	4	Generalist	2	Pupa
Epirrita autumnata	Geometridae		1900	1900	346	84	1	3	Generalist	3	Egg
Acronicta albovenosa	Noctuidae		1900				1	8	Generalist	3	Pupa
Costaconvexa polygram	Geometridae		1900				0	10	Open	2	Pupa
Eilema pygmaeola	Erebidae		1900				0	3	Open	3	Larva
Gastropacha quercifolia	Lasiocampidae		1900				0	5	Forest	3	Larva
Idaea humiliata	Geometridae		1900				0	5	Open	2	Larva
Lemonia dumi	Brahmaeidae		1900				0	4	Open	3	Larva
Abrostola asclepiadis	Noctuidae		1935				0	6	Generalist	1	Pupa
Acontia trabealis	Noctuidae		1935				0	8	Open	2	Pupa
Acronicta strigosa	Noctuidae		1935				1	4	Forest	3	Pupa
Callimorpha dominula	Erebidae		1935				1	3	Forest	3	Larva
Catarhoe rubidata	Geometridae		1935				0	5	Open	2	Pupa
Catocala fulminea	Erebidae		1935				1	4	Generalist	3	Egg
Cerastis leucographa	Noctuidae		1935				0	6	Forest	3	Pupa
Cucullia artemisiae	Noctuidae		1935				0	5	Generalist	2	Pupa
Cucullia fraudatrix	Noctuidae		1935				0	6	Generalist	2	Pupa
Cucullia lychnitis	Noctuidae		1935				0	7	Generalist	1	Pupa
Cucullia scrophulariae	Noctuidae		1935				0	7	Generalist	1	Pupa
Cucullia verbasci	Noctuidae		1935				0	6	Generalist	1	Pupa
Deltote deceptoria	Noctuidae		1935				0	6	Forest	2	Pupa
Dysauxes ancilla	Erebidae		1935				0	3	Open	3	Larva
Eilema griseola	Erebidae		1935				0	7	Forest	3	Larva
Elaphria venustula	Noctuidae		1935				0	5	Forest	3	Larva
Eublemma minutata	Erebidae		1935				0	5	Open	1	Pupa
Euphyia biangulata	Geometridae		1935				0	7	Forest	2	Pupa
Eupithecia orphnata	Geometridae		1935				1	5	Open	3	Pupa
Euproctis chrysorrhoea	Erebidae		1935				0	4	Generalist	3	Larva
Euxoa vitta	Noctuidae		1935				0	6	Open	3	Larva
Hadena compta	Noctuidae		1935				0	8	Generalist	2	Pupa
Hadena filograna	Noctuidae		1935				0	6	Open	1	Pupa
Hadena irregularis	Noctuidae		1935				0	5	Open	1	Pupa
Harpyia milhauseri	Notodontidae		1935				0	7	Forest	2	Pupa
Heliothis viriplaca	Noctuidae		1935				0	10	Open	1	Pupa
Hydraecia nordstroemi	Noctuidae		1935			46	1	8	Generalist	3	Egg
Hyles euphorbiae	Sphingidae		1935				0	5	Generalist	1	Egg
Lacanobia w-latinum	Noctuidae		1935				0	4	Generalist	2	Pupa
Lithostege griseata	Geometridae		1935				0	5	Open	2	Pupa
Lycia pomonaria	Geometridae		1935				0	3	Generalist	3	Pupa
Mythimna albipuncta	Noctuidae		1935				0	9	Open	3	Larva
Nycteola svecicus	Nolidae		1935				0	15	Forest	2	Imago
Pachetra sagittigera	Noctuidae		1935				0	6	Generalist	3	Larva
Pelosia muscerda	Erebidae		1935				0	6	Forest	3	Larva
Pseudeustrotia candidu	Noctuidae		1935				0	6	Generalist	3	Pupa
Pygaera timon	Notodontidae		1935				0	5	Forest	2	Pupa
Sabra harpagula	Drepanidae		1935				0	5	Forest	1	Pupa
Scopula decorata	Geometridae		1935				0	7	Open	1	Larva
Scopula ornata	Geometridae		1935				0	12	Open	2	Larva
Scotopteryx luridata	Geometridae		1935				1	7	Open	1	Larva
Setina roscida	Erebidae		1935				1	6	Open	3	Larva
Spiris striata	Erebidae		1935				1	5	Open	1	Larva
Spodoptera exigua	Noctuidae		1935				0	10	Generalist	3	Larva
Tyta luctuosa	Noctuidae		1935				0	9	Open	2	Larva
Sideridis turbida	Noctuidae		1942				0	7	Generalist	3	Pupa
Hydrelia sylvata	Geometridae		1964				0	5	Forest	2	Pupa
Oligia versicolor	Noctuidae		1966				0	7	Forest	2	Larva
Acossus terebra	Cossidae		1974				0	2	Forest	1	Larva
Heliothis peltigera	Noctuidae		1975				0	16	Generalist	3	Pupa
Selidosema brunnearia	Geometridae		1975				1	5	Open	2	Larva
Theria rupicapraria	Geometridae		1975				0	3	Open	2	Pupa
Rhodometra sacraria	Geometridae		1983				1	6	Generalist	3	Larva
Noctua janthina	Noctuidae		1984			5	0	4	Generalist	3	Larva
Phragmataecia castane	Cossidae		1984				0	5	Generalist	2	Larva
Horisme vitalbata	Geometridae		1985				0	6	Open	2	Pupa
Pelosia obtusa	Erebidae		1986				0	3	Generalist	1	Larva
Noctua interposita	Noctuidae		1991			2	1	7	Generalist	3	Larva
Apamea epomidion	Noctuidae		1992				0	8	Forest	2	Larva
Cirrhia ocellaris	Noctuidae		1992				1	5	Generalist	1	Egg
Proxenus lepigone	Noctuidae		1994				0	4	Generalist	1	Larva
Grammodes stolida	Erebidae		1996				0	12	Generalist	3	Egg
Thaumetopoea pinivora	Notodontidae		1996				0	7	Forest	2	Larva
Pabulatrix pabulatricula	Noctuidae		1997				0	4	Forest	3	Egg
Athetis gluteosa	Noctuidae		2002				0	4	Generalist	1	Larva

Colobochyla salicalis	Erebidae		2003				0	4	Generalist	2	Pupa
Heterogenea asella	Limacodidae		2003				0	7	Forest	2	Pupa
Meganola albula	Nolidae		2004				1	6	Generalist	3	Larva
Mythimna unipuncta	Noctuidae		2004				0	8	Generalist	3	Egg
Sedina buettneri	Noctuidae		2004				0	4	Generalist	1	Egg
Deltote bankiana	Noctuidae		2005			1	0	7	Open	2	Pupa
Schrankia taenialis	Erebidae		2006				0	3	Forest	2	Larva
Hippotion celerio	Sphingidae		2010				0	12	Generalist	3	Egg
Calyptra thalictri	Erebidae		2014				0	5	Forest	1	Larva
Agrotis puta	Noctuidae		2016				2	8	Generalist	3	Larva
Catocala elocata	Erebidae		2016				0	6	Forest	2	Egg
Cyclophora porata	Geometridae		2016				1	8	Forest	2	Pupa
Xanthorhoe annotinata	Geometridae		2016				0	5	Forest	2	Larva
Cryphia algae	Noctuidae		2017			118	1	5	Generalist	3	Larva
Cyclophora annularia	Geometridae		2017				0	4	Generalist	1	Pupa
Idaea trigeminata	Geometridae		2017				0	4	Generalist	3	Larva
Thetidia smaragdaria	Geometridae		2017				0	4	Open	2	Larva
Caradrina kadenii	Noctuidae		2019				1	18	Generalist	3	Pupa
Eupithecia pulchellata	Geometridae		2019				0	4	Open	1	Pupa
Mythimna vitellina	Noctuidae		2019				0	10	Generalist	3	Pupa
Conisania luteago	Noctuidae		2020			1	0	8	Generalist	3	Pupa
